# Sarcopenia: the need for early detection, early treatment, and the identification of sensitive measures for predicting treatment response

**DOI:** 10.2478/abm-2025-0001

**Published:** 2025-02-28

**Authors:** Editorial Office of Asian Biomedicine

**Affiliations:** Faculty of Medicine, Chulalongkorn University, Bangkok 10330, Thailand

Older people usually have reduced muscle mass, muscle strength, and a decline in physical functioning, which finally give rise to reduced functionality. The progressive loss of muscle mass and function can lead to frailty, functionality, falls, fracture, physical disability, reduced quality of life, and eventual mortality [1]. The concurrent loss of muscle mass together with muscle strength occurs as a result of the decline in skeletal muscle cells and functions leading to adverse health outcomes called sarcopenia [1]. The European Working Group on Sarcopenia in Older People (EWGSOP) developed a practical clinical definition and consensus diagnostic criteria for age-related sarcopenia. EWGSOP included representatives from four participant organizations, including the European Geriatric Medicine Society, the European Society for Clinical Nutrition and Metabolism, the International Association of Gerontology and Geriatrics and the European Region, and the International Association of Nutrition and Aging [2, 3].

It is essential for older people who seek care because of weakness to have a detailed history and physical examination to document whether secondary cause of sarcopenia is present and whether the weakness affects their basic activities of daily living. A thorough history can also document the onset, progression of weakness, associated medical conditions (endocrine disorders, electrolyte disturbance, malabsorption, and other systemic diseases), medications, adequate nutrition, and obesity. A history of family members with muscle weakness can be helpful to check for possible genetic linkages, particularly if the patients come with chronic muscle disorders [1].

Understanding sarcopenia's etiology and consequences is crucial for healthcare professionals to enhance patient outcomes. In this issue, Manjavong et al. [4] reported a relatively low awareness of sarcopenia among trainee physicians. Furthermore, knowledge regarding sarcopenia case finding, screening, diagnosis, and treatment strategy is still superficial among university-based physicians. Although trainees' limited knowledge is emphasized in this short communication, researchers and clinicians should focus on sarcopenia biological pathway. This is important for early detection, treatment, and identification of sensitive measures for predicting sarcopenia treatment response [5].

Effective management of sarcopenia requires a systematic identification of the underlying causes and managing accordingly. Most people with documented sarcopenia will need nutritional support, and exercise interventions. Recent research emphasizes pharmacological treatments targeting hormonal and metabolic changes [5, 6]. Cellular and animal models have generated some evidence that supports possible beneficial effects on skeletal muscle function for some classes of drugs used to treat diabetes, including metformin and sodium-glucose transport protein 2 inhibitors [6].
